# CD105+CAF-derived exosomes CircAMPK1 promotes pancreatic cancer progression by activating autophagy

**DOI:** 10.1186/s40164-024-00533-3

**Published:** 2024-08-05

**Authors:** Zhiwei He, Xiushen Li, Shiyu Chen, Kun Cai, Xiaowu Li, Hui Liu

**Affiliations:** 1 Department of Hepatobiliary Surgery, The Affiliated Hospital of Guizhou Medical University, Guizhou Medical University, Guiyang, 550001 People’s Republic of China; 2 Department of Hepatobiliary Surgery, Shenzhen University General Hospital & Shenzhen University Clinical Medical Academy Center, Shenzhen University, Shenzhen, 518000 Guangdong People’s Republic of China; 3 Guangdong Key Laboratory for Biomedical Measurements and Ultrasound Imaging, School of Biomedical Engineering, Shenzhen University Health Science Center, Shenzhen, 518060 People’s Republic of China; 4 Department of Hepatic-Biliary-Pancreatic Surgery, South China Hospital, Medical School, Shenzhen University, Shenzhen, 518116 People’s Republic of China

## Abstract

Previous studies have shown that the heterogeneity of tumor-associated fibroblasts (CAFs) in the tumor microenvironment may play a critical role in tumorigenesis; however, the biological function of CAFs in pancreatic cancer is still controversial. In this study, we found that CD105-positive (CD105^+^) CAF-derived exosomes significantly promoted the proliferative and invasive metastatic abilities of pancreatic cancer cells. Furthermore, RNA-seq and qRT‒PCR experiments revealed circAMPK1 as a key molecule in exosomes from CD105^+^ CAFs that mediates the malignant progression of pancreatic cancer. Furthermore, we demonstrated that circAMPK1 encodes a novel protein (AMPK1-360aa) in pancreatic cancer cells. This protein competes with AMPK1 to bind to the ubiquitination ligase NEDD4, which inhibits AMPK1 protein degradation and ubiquitination and thereby increases AMPK1 levels. Finally, we demonstrated that AMPK1-360aa induces cellular autophagy via NEDD4/AMPK1 to promote the proliferation and invasion of pancreatic cancer cells. In summary, circAMPK1 in CD105^+^ CAF-derived exosomes may mediate pancreatic cancer cell proliferation and invasive metastasis by inducing autophagy in target cells. Moreover, circAMPK1 may competitively bind to ubiquitinating enzymes through the encoded protein AMPK1-360aa, which in turn inhibits the ubiquitination-mediated degradation of AMPK1 and contributes to the upregulation of AMPK1 expression, thus inducing cellular autophagy to mediate the malignant progression of pancreatic cancer.

**Supplementary Information:**

The online version contains supplementary material available at 10.1186/s40164-024-00533-3.

## Background

Pancreatic cancer (PC), which is a highly aggressive malignant gastrointestinal tumor, has a low incidence but high mortality rate, with a 5-year survival rate of approximately 8% [1]. The vast majority of patients are diagnosed with advanced and inoperable PC, and an increasing number of studies have shown that the complex ecosystem in the tumor microenvironment (TME) may be a key factor that mediates the drug resistance and tumor progression of PC [2–6]. The TME consists mainly of tumor cells, infiltrating immune cells (e.g., macrophages), cancer-associated stromal cells (e.g., cancer-associated fibroblasts [CAFs]), endothelial cells, and several other cell populations [7, 8]. CAFs, which are some of the most important components of the TME, are thought to be involved in biological processes such as the immune escape, proliferation, metastasis, and angiogenesis of PC cells [9–12]. In a mouse model of PC, the absence of α-SMA + CAFs has been shown to accelerate tumor progression, reduce the fibrotic response and decrease survival. In addition, clinical sample testing has suggested that higher tumor α-SMA levels and mesenchymal densities correlate with good overall survival outcomes in PC patients [13]. These findings suggest that different CAF subgroups may be present in PC. A recent panoramic analysis of CAFs in PC tissues from KPC mice revealed that CD105^+^ CAFs promote tumor cell proliferation to increase tumor resistance, whereas CD105^−^ CAFs play a tumor-suppressive role by inducing tumor immunity [14]. These findings suggest that CD105^+^ CAFs may be a key cell subpopulation that promotes the malignant progression of PC; however, the molecular mechanisms of these cells remain unknown.

Previous research has suggested that exosomes may be key carriers of signaling molecules by which CAFs interact with cancer cells; thus, studies on the regulation of biological behaviors of cancer cells by exosomes have gained widespread attention in recent years. A large body of evidence suggests that exosomes can play a procancer role as mediators of intercellular communication in the TME [15, 16]. Exosomes are small, single-membrane vesicles with a diameter of 30–100 nm that are released by various cells. Exosomes usually contain substances such as circRNAs, lncRNAs and proteins, which play key roles in cellular communication [17–19]. CAFs in breast cancer tissues can deliver circTBPL1 to tumor cells via exosomes and exert procarcinogenic effects through the miR-653-5p/TPBG pathway [20]. The downregulation of miR-501 by an exosome inhibitor (GW4869) or miR-501 inhibitor was found to restore the sensitivity of chemotherapy-resistant cells to adriamycin and to inhibit the proliferation, migration, invasion and apoptosis of gastric cancer cells [21]. In the presence of chemotherapeutic agents, increased secretion of CAF exosomes is observed in PC, along with significant changes in the levels of exosome cargoes, all of which ultimately induce the proliferation and metastasis of tumor cells [22].

CircRNAs are a specific type of noncoding RNA; they are formed by alternative splicing and have a loop structure that their degradation difficult. CircRNAs are specifically expressed in tumors and serum exosomes and are often used as biomarkers of tumorigenesis and progression [23–27]. In terms of molecular mechanisms, circRNAs promote tumor progression mainly by adsorbing tumor cells [28]. CircRNAs can act as protein decoys or scaffolds to recruit proteins such as NEDD4 to mediate changes in the ubiquitination activity of target genes, thus influencing cancer progression [29]. However, recent studies have revealed that circRNAs can encode peptides and proteins. For example, circCUX1 encodes the p113 subunit, which in turn forms a transcriptional regulatory complex with ZRF1 and BRD4 to activate lipid metabolism to promote the malignant progression of tumors [30]. CircFBXW7 encodes a novel protein (FBXW-185aa), which inhibits the activity of USP28 to mediate c-MYC degradation, thus ultimately inhibiting the malignant progression of gliomas [31]. CircSHPRH inhibits the proteasomal degradation of SHPRH by competitively binding to ubiquitinating enzymes, which in turn inhibits glioma proliferation [32]. Furthermore, circAXIN1 encodes AXIN1-295aa, which can competitively bind to APC to prevent the formation of the AXIN1-APC-GSK3B complex and activate the β-catenin signaling pathway to promote gastric cancer progression [33].

In the present study, we revealed that a novel circRNA, circAMPK1, in CD105^+^ CAF-derived exosomes (CAF-Exos) is a key molecule that may promote PC progression. We also discovered via bioinformatics and molecular biology analyses that circAMPK1 encodes the novel protein AMPK1-360aa. AMPK1-360aa competitively binds to NEDD4, thereby inhibiting the ubiquitination-mediated degradation of AMPK1 and contributing to the upregulation of its expression. This, in turn, induces cellular autophagy to promote PC progression. The findings of this study are expected to provide new theories for PC research, as well as new targets and approaches for PC treatment.

## Methods

### Human tumor tissues and cell lines

A total of 120 normal pancreatic tissues and PC samples were collected from the Department of Hepatobiliary Surgery of The Affiliated Hospital of Guizhou Medical University and Shenzhen University General Hospital, with written informed consent from the patients. The Clinical Research Ethics Committee authorized the project. All of the experimental techniques complied with the Declaration of Helsinki. Pancreatic cancer cell lines were obtained from the American Type Culture Collection. The cell culture media included RPMI 1640 medium (Gibco, USA) and DMEM supplemented with 10% fetal bovine serum (FBS) (Gibco, USA), 100 μg/mL streptomycin, and 100 IU/mL penicillin. All of the cell lines were maintained at 37 °C in a humidified incubator with 5% CO2.

### Animal care and ethics statement

Beijing Vital River Laboratory Animal Technology Co., Ltd., provided four-week-old female BALB/c-nu mice. The mice were kept in a specific pathogen-free facility with free access to food and water under controlled temperature and light conditions. The Institutional Animal Care and Use Committee of Shenzhen University approved all of the experimental methods involving the handling of mice.

### Animal experiments

The PC liver metastasis model that was used in this study was established as previously described. The BALB/c nude mice that were used in the experiments were 4-week-old female mice. The cell mixture (200 μL) was subcutaneously injected into the right armpits of BALB/c nude mice to establish the subcutaneous transplantation model. Using a caliper, tumor growth was monitored, and the tumor volume was computed using the formula volume (mm^3^) = LW^2^/2, where L represents length (mm) and W represents width (mm). After 6 weeks, the mice were euthanized, and their subcutaneous tumors were removed and preserved in formaldehyde for hematoxylin and eosin (H&E) staining.

### Western blotting and antibodies

Western blotting was performed as previously reported [34]. The primary antibodies that were used for Western blotting and immunofluorescence of CD63, AMPK1, NEDD4, ULK1, LC3, P62, HA, and Flag were purchased from Abcam. An Integrin Antibody Sampler Kit and a GAPDH antibody were purchased from Cell Signaling. All of the secondary antibodies, including polyclonal goat mouse and goat rabbit IgG, were HRP-conjugated and were acquired from Cell Signaling. An imaging system (Thermo Fisher) and the program ImageJ were used to measure the relative expression levels.

### Plasmids and transfection

Chemical gene synthesis was used to construct a circAMPK1 overexpression plasmid, a circAMPK1-3xFlag plasmid, a 360aa-3xFlag plasmid, a mutSD plasmid, a circ-frame Del plasmid, a circAMPK1 noATG plasmid, and a circAMPK1 Mut plasmid. The EMCV-IRES sequence, the putative circAMPK1 internal ribosome entry site (IRES) sequence, and the IERS-Delete sequences were chemically synthesized and inserted into the Circ-RLuc-IRES-Reporter vector between the “uc” and “RL” sequences to validate IRES activity. Following the manufacturer's instructions, Lipofectamine 3000 (Invitrogen, Carlsbad, CA, USA) was used to transfect the plasmids.

### Exosome isolation

Pancreatic cancer cells were cultured in complete medium supplemented with exosome-free FBS for 48 h. The supernatants were collected and then subjected to a series of differential centrifugation steps to remove intact cells and cellular debris. The supernatants were transferred and centrifuged. After discarding the supernatants, the exosomes were washed with PBS and centrifuged at 100,000×*g* for another 70 min to purify the exosomes.

### Immunohistochemistry (IHC)

All of the paraffin-embedded tumor sections were deparaffinized and blocked. Primary antibodies were administered overnight at 4 °C in a wet chamber after being diluted in bovine serum albumin. Following secondary antibody incubation, the tumor slices were subsequently treated with diaminobenzidine reagent and counterstained with hematoxylin. Based on the staining intensity and the percentage of positive cells, we quantitatively assessed the tumor tissue sections.

### Coimmunoprecipitation and mass spectrometry analysis

Lysates from PANC-1 cells transfected with FLAG-circAMPK1 were prepared. FLAG and IGG antibodies were added separately, and the lysates were incubated overnight at 4 °C. Affinity agarose beads were added to the lysate and shaken vertically for four hours. Afterward, the beads were washed five times using prechilled immunoprecipitation (IP) wash solution. After being heated and denatured, the eluted proteins were subjected to gel electrophoresis and Coomassie blue staining. Mass spectrometry was used to examine the bands corresponding to the target proteins of interest.

### Immunofluorescence

After being fixed for 10 min with 4% formaldehyde, the cultured cells were blocked for 30 min at room temperature using 5% BSA and 0.1% Triton X-100 in PBS. The relevant primary and secondary antibodies were used for immunostaining. The counterstain that was used on the nuclei was 4,6-diamidino-2-phenylindole (DAPI).

### RNA sequencing (RNA-seq) analysis and identification of circRNAs

SEQHEALTH used an Illumina HiSeq™ 2500 to perform RNA-seq. The reads were mapped to a ribosomal RNA (rRNA) database using the short-read alignment tool Bowtie2. The reads that mapped to rRNA were eliminated. Alignment and analysis were conducted using the remaining reads. After the reads that could be mapped to the genome were discarded, the unmapped reads were collected for circRNA identification. If at least two distinct back-spliced reads were present in at least one sample, they were considered to represent a candidate circRNA. The circRNAs were annotated with circBase. CircRNAs that exhibited a fold change ≥ 2 and P value < 0.05 when compared between samples or groups were considered to be significantly differentially expressed.

### CAF or normal fibroblast (NF) isolation and culture

CAFs and primary NFs were isolated from PC tissues and corresponding noncancerous tissues. Freshly isolated surgical resection specimens were collected with informed consent from Shenzhen University General Hospital. The core portion of the PC tissue was minced with a sterile scalpel, and the tissue was digested with collagenase digestion medium for 30 min and quenched in 10% fetal bovine serum/DMEM. The isolated tissue was then incubated in a 6-cm Petri dish at 37 °C for 10 min without shaking. The supernatants, which were enriched with mesenchymal cells, were collected in new tubes and centrifuged at 250×*g* for 5 min.

### CCK-8 assay

Individual wells of a 96-well plate were seeded with the specified cells. Every 24 h, the absorbance of the cells was measured at 450 nm using CCK-8 reagent (Dojindo) following the manufacturer's instructions to assess cell viability.

### Wound-healing assay

Various groups of cells were cultured in DMEM supplemented with 10% FBS in 6-well plates, and after 80–90% confluent monolayers formed, wound healing assays were performed. The cell monolayers were scratched vertically using a 200 μl pipette tip. After being washed three times with PBS, the cells were incubated in serum-free medium for 48 h. The wound width was then measured.

### Transwell assay

Cells (200 μl, 2 × 10^5^/ml) in DMEM were placed in the upper chamber of a Transwell device with 10% FBS medium in the bottom chamber. Subsequently, the cells were allowed to migrate under standard culture conditions for one day. The cells that migrated were fixed with 4% paraformaldehyde for 30 min and stained with crystal violet. The cells that did not move through the top chamber were removed with a cotton swab. For the invasion assay, the upper chamber of the Transwell was coated with Matrigel before the cells were seeded.

### Actinomycin D assay

PC cells were evenly seeded into five wells in 6-well plates. The cells were then treated with actinomycin D (2 μg/ml) for 0, 4, 8, 12, or 24 h. Subsequently, the cells were isolated, and the relative RNA levels of linear AMPK1 and circ-AMPK1 were examined via qRT‒PCR. The results were normalized to those recorded in the 0 h group.

### RNA subcellular isolation

Using an RNA subcellular separation kit (Active Motif, Inc., Carlsbad, CA, USA), the cytoplasmic and nuclear fractions were separated. Briefly, cells were lysed in complete lysis solution and allowed to sit on ice for ten minutes. Following centrifugation, the remaining pellet was collected for nuclear RNA purification, and the supernatant was removed for cytoplasmic RNA extraction. The RNA products were subjected to qRT‒PCR.

### Organoid culture

Clinical tissue specimens were collected, chopped into pieces of approximately 0.5–1 mm3, digested into single-cell suspensions with digestive solution containing 2.5 mg/ml collagenase D and 0.1 mg/ml DNase I, incubated at 37 °C for 30 min, digested, filtered, added to medium and resuspended using a microfluidic device to prepare single drops of a cell suspension. A single drop was added to each well of 24- or 48-well plates with a single drop of stromal gel. The plates were incubated at 37 °C for 5–10 min until the mixture of cell suspension and stromal gel was fixed. The mixture was then added to expansion medium and cultured for 3–4 days. The organoids were visible after 14 days of culture. The other cells were incubated for 5–10 min with matrix gel until the mixture was fixed. The isolation medium was subsequently added, and the cells were incubated for 3–4 days. Afterward, the medium was changed to expansion medium, and the organoids were observed after 7 days of incubation and passaged before 14 days of incubation.

### Statistics

The findings are shown as the mean plus standard deviation (SD). To evaluate the differences between the experimental and control groups, Student's t test was used. To analyze correlations in various specimens, Pearson's test was employed. The Kaplan–Meier test was used for overall survival (OS) and disease-free survival (DFS) analyses. All of the experiments were independently performed twice.

## Results

### ***CD105***^+^***CAF-derived exosomes promote proliferation and metastasis in PC***

**Fig. 1:**
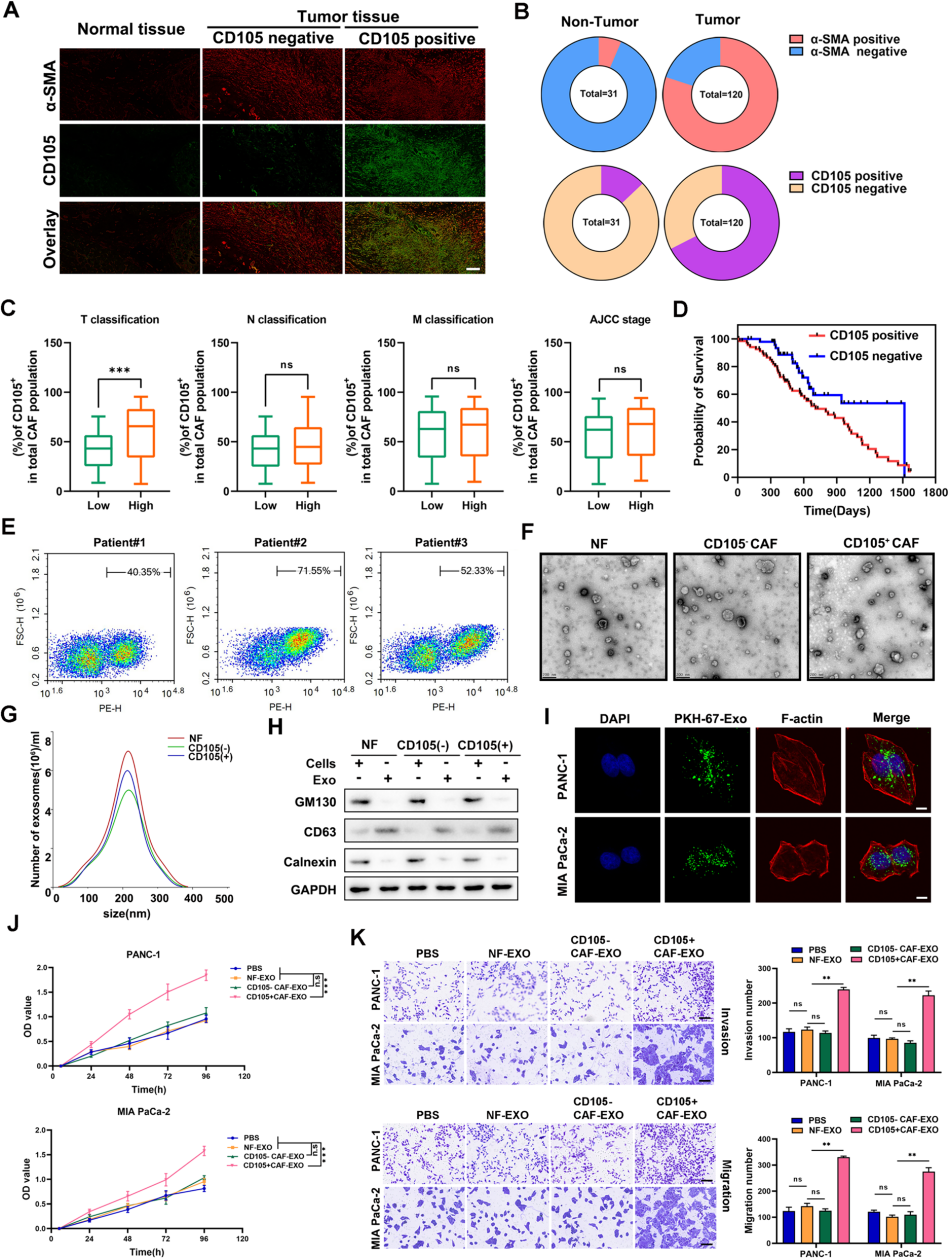
Exosomes from CD105^+^ CAFs promote the proliferation and metastasis of PC cells. **A**, **B** Immunofluorescence assays were used to detect α-SMA and CD105 in normal adjacent tissue and tumor tissues. **C** Correlation of the proportion of CD105^+^ CAFs of patients with T, M, N, and AJCC stages PC. **D** Survival curve analysis of CD105^+^ and CD105^−^ patient prognosis. **E** CD105-positive CAFs in PC tissues were analyzed using flow cytometry. **F**–**H** Electron microscopy and particle size were used to analyze the morphology and diameter distribution of the exosomes, and immunoblotting was used to detect exosomal markers. **I** Immunofluorescence staining showing the internalization of CAF-derived exosomes into PC cells. **J** Effects of NF-Exos, CD105^−^ CAF-Exos, and CD105^+^ CAF-Exos on PC cell proliferation, as measured via CCK-8 assay. **K** Effects of NF-Exos, CD105^−^ CAF-Exos, and CD105^+^ CAF-Exos on the proliferation of PC cells, as measured via Transwell metastasis and invasion assays

To observe the numbers of CD105^+^ CAFs in PC, we collected 120 PC tissues and 31 adjacent normal tissues. The results of immunofluorescence experiments showed that a greater proportion of CD105^+^ CAFs were present in tumor tissues than in normal tissues (Fig. 1A, B). In addition, the proportion of CD105^+^ CAFs was positively correlated with the T stage of PC and with poor patient prognosis (Fig. 1C, D). Furthermore, we isolated NFs, CD105^−^ CAFs and CD105^+^ CAFs and cocultured them with PC cells (Fig. 1E, Additional file 1: Fig. S1A). CD105^+^ CAFs significantly promoted the proliferation, invasion and migration of PC cells (Additional file 1: Fig. S1B–S1D). Exosomes were identified by transmission electron microscopy, particle size analysis and Western blotting. The results suggested that the exosomes had a characteristic cup-shaped structure with diameters that were mostly in the range of 100–300 nm and that they specifically expressed CD63 (Fig. 1F–H). PC cells were incubated with exosomes secreted by CD105^+^ CAFs, and immunofluorescence staining showed that the exosomes were able to enter the PC cells (Fig. 1I). PBS, NF-Exos, CD105^+^ CAF-Exos and CD105- CAF-Exos were added to PC cells for culture, and the results showed that CD105^+^ CAF-Exos promoted the proliferation and metastasis of PC cells (Fig. 1J, K and Additional file 2: Fig. S2A-S2B). These results indicate that CD105^+^ CAF cell-derived exosomes promote proliferation and metastasis in PC.

### ***Characterization and analysis of circAMPK1 expression in exosomes from CD105***^+^***CAFs in PC***

**Fig. 2:**
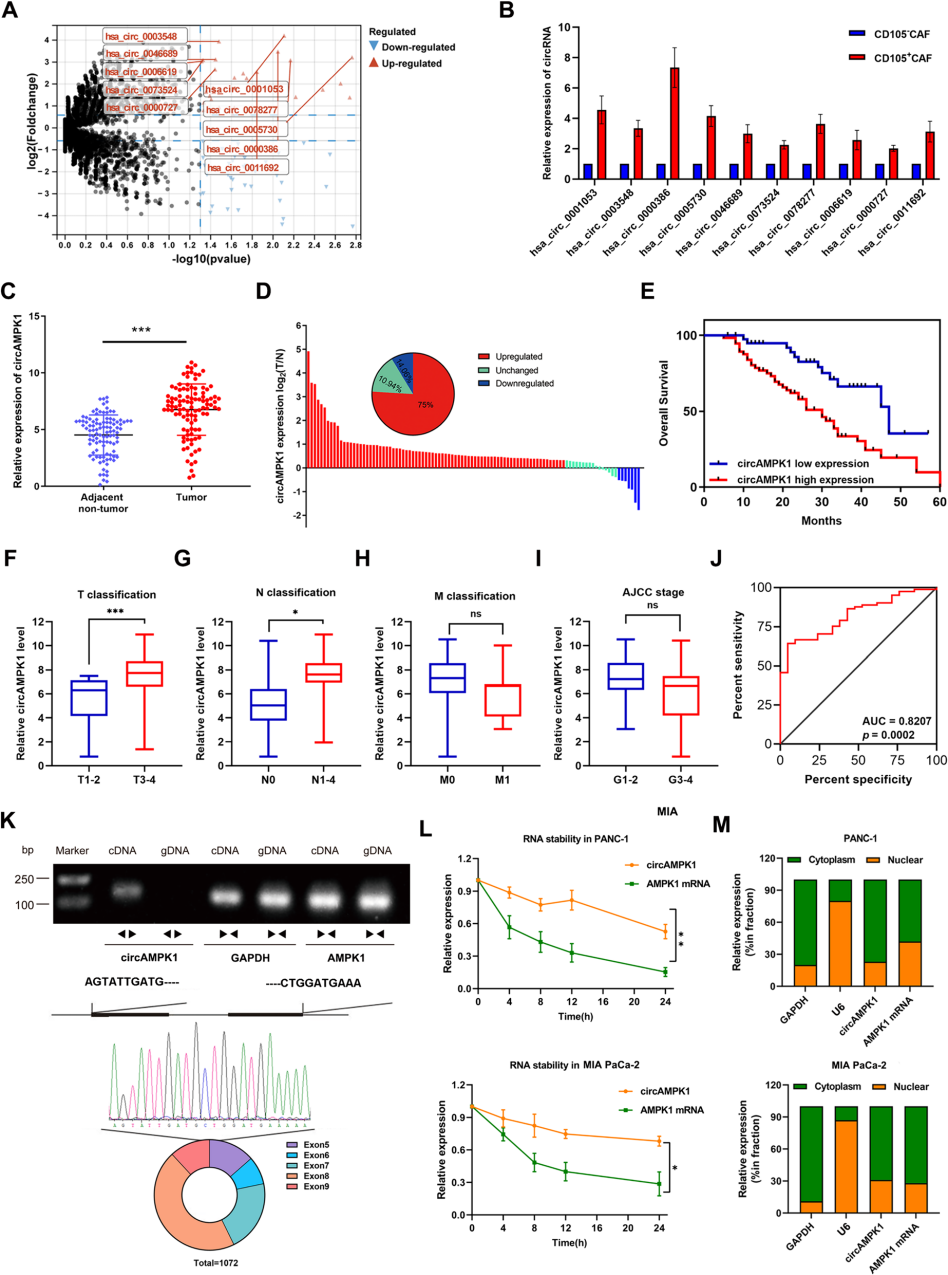
Characterization and expression of circAMPK1 in the exosomes of CD105 + CAFs in PC. **A** Differential circRNA expression in the exosomes of CD105^+^ and CD105^−^ CAFs was analyzed using RNA-seq. **B** The top 10 circRNAs with substantially different expression levels were confirmed by qRT‒PCR. **C** The expression level of circAMPK1 in PC paracarcinoma and tumor tissues was determined using qRT‒PCR. **D** Box-and-line plots showing the expression of circAMPK1 in paracancerous and PC tumor tissues. **E** Survival analysis of patients with different expression levels of circAMPK1. **F**–**I** Correlations between circAMPK1 expression and PC T, N, M, and AJCC stage. **J** AUC analysis of the efficacy of circAMPK1 expression as a PC biomarker. **K** Upper panel: CircAMPK1 was found in cDNA but not in gDNA according to a qRT‒PCR experiment using divergent or convergent primers. GAPDH served as a negative reference point. Lower panel: Head-to-head tail splicing of circAMPK1 validated via Sanger sequencing. **L** qRT‒PCR analysis of the RNA stability of circAMPK1 and AMPK1 mRNA after the treatment of PC cells with actinomycin D for different durations. **M** qRT‒PCR confirming that circAMPK1 and AMPK1 mRNAs are located mainly in the cytoplasm of PC cells

To identify the key circRNAs in CD105^+^ CAF-Exos that promote the proliferation and metastasis of PC cells, we subjected CD105^+^ CAF and CD105^−^ CAFs to transcriptome sequencing (Fig. 2A, Additional file 5: Table S1, Additional file 6: Table S2). qRT‒PCR was used to validate the 10 candidate circRNAs with the most significant upregulation, and we found that hsa_circ_0003548 (circAMPK1) was the circRNA that exhibited the most significant difference (Fig. 2B). Comparison of circAMPK1 expression in PC clinical samples and matched paracancerous samples via qRT‒PCR revealed that circAMPK1 was similarly substantially expressed in PC clinical samples (Fig. 2C, D). Survival curve analysis revealed that patients with high circAMPK1 expression had a worse prognosis (Fig. 2E). circAMPK1 expression was positively correlated with PC T and N stage but not significantly correlated with M stage or the AJCC stage (Fig. 2F–I). The area under the receiver operating curve (AUC) analysis suggested that high circAMPK1 expression is a biomarker of PC (Fig. 2J). Using junction-specific primers, we amplified only circAMPK1 from random-primer reverse-transcribed cDNA but not from oligo-dT reverse-transcribed cDNA (Fig. 2K). Compared with AMPK1 mRNA, circAMPK1 had a longer half-life (Fig. 2L). Nucleoplasmic separation experiments showed that AMPK1 was mostly observed in the nucleus, whereas circAMPK1 was primarily found in the cytoplasm (Fig. 2M). After the incubation of PC cells with PBS, sh-Control-Exos, sh-circAMPK1#1-Exos, or sh-circAMPK1#2-Exos, we found that silencing circAMPK1 in CD105^+^ CAFs partially reversed the effects of exosomes on the proliferation and invasion of PC cells (Additional file 3: Fig. S3A-S3B). These results demonstrated that circAMPK1 is the key circRNA in CD105^+^ CAF-derived exosomes that mediates PC cell proliferation and metastasis.

### circAMPK1 encodes a novel 360-amino acid (aa) protein known as AMPK1-360aa

**Fig. 3:**
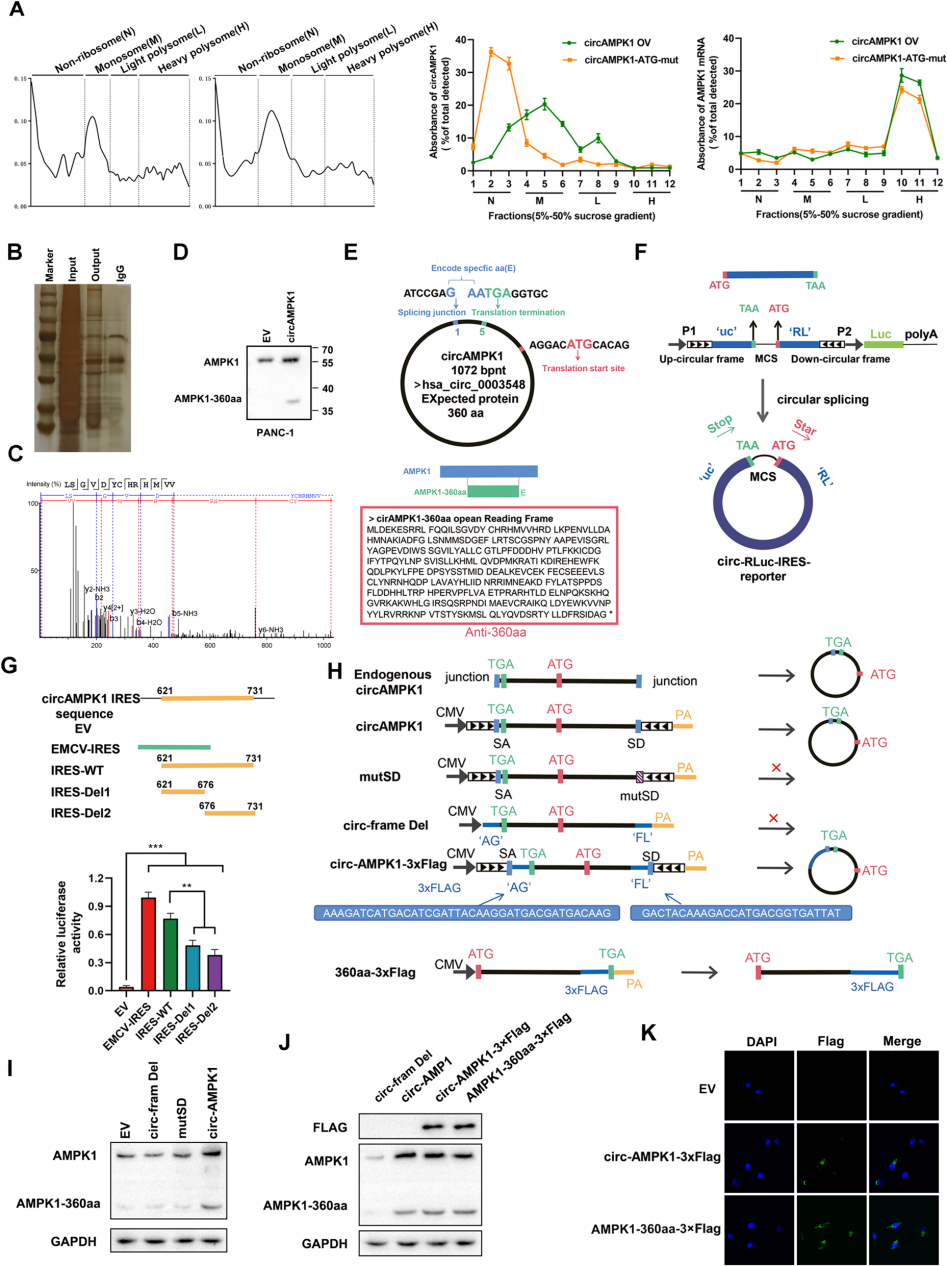
CircAMPK1 encodes a 360-aa novel protein known as AMPK1-360aa. **A** Sucrose gradient (5–50%) ultracentrifugation was used to separate the polysome fractions (N, nonribosome; M, monosome; L, light polysome; and H, heavy polysome) from circ_AMPK1- or circ_AMPK1-ATG-mut-overexpressing 293T cells. The translation potential of circ_AMPK1 was then examined by qRT‒PCR with junction-specific primers. As a positive control, qRT‒PCR was performed utilizing primers unique to linear AMPK1 to determine its translation capacity. **B**, **C** After 293T cells were transfected with vector or FLAG-circ_AMPK1, total protein was isolated, and the AMPK1-360aa peptide sequences were identified via LC‒MS analysis. **D** AMPK1-360aa was detected in cells overexpressing circAMPK1 via Western blotting. **E** Bioinformatics analysis of the amino acid sequence of AMPK1-360aa. **F**, **H** Construction of exogenous circAMPK1 ORF expression vectors. **G** Construction of IRES deletion mutants of exogenous circAMPK1 and verification of the ability of circAMPK1 to bind to ribosomal translation initiation sites via dual-luciferase reporter assay. **H** Construction of overexpression vectors with deletion and mutation of ORFs and assessment of the encoding ability of circAMPK1 in different groups. **I** The expression levels of AMPK1-360aa encoded by circAMPK1 after deletion of the ORF and mutation of the IRES binding site were assessed by Western blotting. **J**, **K** The expression levels of AMPK1-360aa after overexpression of Flag-circAMPK1 or Flag-AMPK1-360aa were assessed by Western blotting and immunofluorescence

Several studies have shown that circRNAs encode proteins and polypeptides, and we predicted that circAMPK1 can be translated. The translatability of circAMPK1 was examined via a sucrose density gradient centrifugation-based multimer assay. In the circAMPK1 overexpression group, circAMPK1 was mainly distributed in the monosome (M) and light polysome (L) compartments, unlike in the circAMPK1 ATG mutation group, in which circAMPK1 was mainly distributed in the nonribosome (N) domain. Linear AMPK1, which was used as a positive control, was mainly distributed in the heavy polysome (H) component in both groups (Fig. 3A). To confirm that circAMPK1 can be translated into AMPK1-360aa, we used an RNA pulldown assay in combination with LC‒MS/MS. We also detected the AMPK1-360aa signal by using Western blotting (Fig. 3B–D). The amino acid sequences of the circAMPK1 open reading frame (ORF) and of AMPK1-360aa were obtained via bioinformatics analysis, and an IRES deletion mutant was constructed based on the prediction results. The results of a dual-luciferase reporter assay confirmed that circAMPK1 could bind to ribosomal translation initiation sites (Fig. 3E–G). Afterward, we found that the coding ability of circAMPK1 disappeared after the ORF was deleted (Fig. 3H). Subsequently, the results of immunoblotting experiments showed that the expression of the encoded product AMPK1-360aa was significantly decreased after deletion of the ORF and mutation of the IRES binding site (Fig. 3H). In contrast, overexpression of circAMPK1 or AMPK1-360aa markedly increased the expression level of the encoded product (Fig. 3I, J). Immunofluorescence showed that both circAMPK1 and its encoded product (AMPK1-360aa) were present in the cytoplasm (Fig. 3K). These results suggest that circAMPK1 can encode a novel AMPK1 variant known as AMPK1-360aa.

### AMPK1-360aa interacts with NEDD4 to inhibit AMPK1 ubiquitination and degradation

**Fig. 4:**
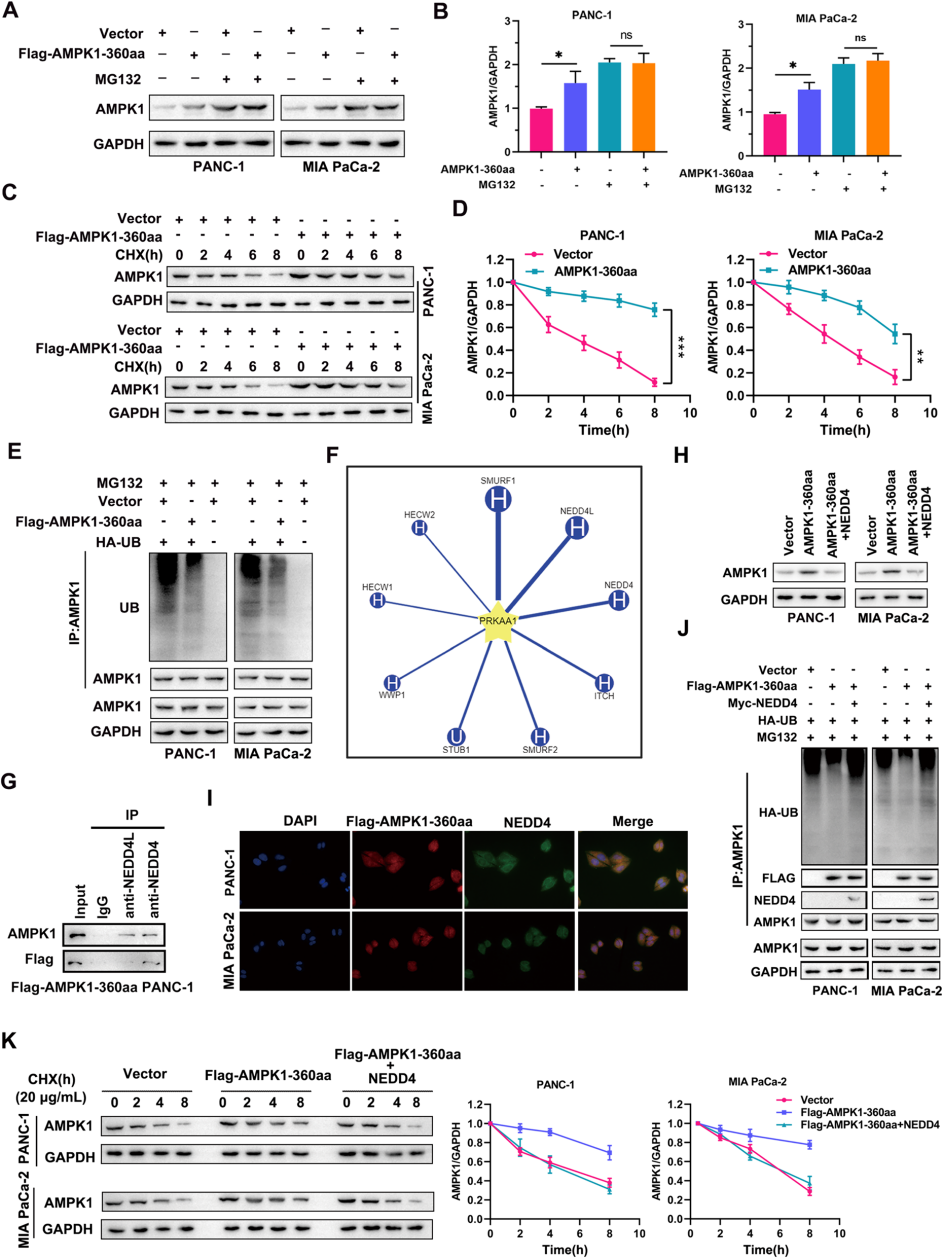
AMPK1-360aa interacts with NEDD4 to inhibit AMPK1 degradation. **A**, **B** The expression levels of AMPK1 in PC cells transfected with empty vector or Flag-AMPK1-360aa and treated with or without MG132 (20 μM) were measured by Western blotting. **C**, **D** To determine the stability of the AMPK1 protein in PC cells transfected with either empty vector or Flag-AMPK1-360aa, Western blotting was conducted, and the cells were exposed to cycloheximide (CHX; 100 μg/ml) for 0, 2, 4, 6, or 8 h. **E** IP analysis of the ubiquitination level of AMPK1 in PC cells transfected with or without Flag-AMPK1-360aa followed by immunoblotting with an anti-ubiquitin antibody. **F** The level of the E3 ubiquitin ligase upstream of AMPK1 was analyzed with the UBIbrowser database. **G** Assessment of whether NEDD4L and NEDD4 can bind to AMPK1 and AMPK1-360aa by IP. **H** To determine whether PC cells transfected with the empty vector, the AMPK1-360aa plasmid, or the AMPK1-360a plasmid + NEDD4 plasmid expressed AMPK1, Western blotting was conducted. **I** Immunofluorescence analysis showing the colocalization of AMPK1-360aa and NEDD4 in PC cells. **J** IP was used to assess the level of AMPK1 ubiquitination in PC cells transfected with Flag-AMPK1-360aa, Myc-NEDD4-transfected AMPK1 protein, or empty vector, and immunoblotting was performed using an anti-ubiquitin antibody. **K** Assessment of AMPK1 protein stability in PC cells transfected with empty vector, Flag-AMPK1-360aa, or Flag-AMPK1-360aa + NEDD4

First, we found that the mRNA expression of AMPK1 in PC cells was not increased by overexpression of AMPK1-360aa, according to the qRT‒PCR results (Additional file 4: Fig. S4A). Therefore, we hypothesized that differential AMPK1 expression occurs as a result of protein degradation associated with autophagy in lysosomes and proteasomes. In PC cell lines, we examined the expression of AMPK1, and the results indicated that AMPK1 was significantly highly expressed in PANC-1 and Mia PaCa-2 cells (Additional file 4: Fig. S4B). We blocked autophagy by adding the autophagy inhibitors 3-methyladenine (3MA), bafilomycin A1 (Baf), and chloroquine (CQ) to the cells and blocked proteasome activity by adding MG132. We found that the expression of AMPK1 was significantly elevated in the AMPK1-360aa overexpression group but not in the proteasome inhibitor group (Fig. 4A, B). However, AMPK1 expression was still upregulated after autophagy was blocked (Additional file 4: Fig. S4C-S4E). Protein stability assays suggested that AMPK1 stability was significantly increased in the AMPK1-360aa overexpression group (Fig. 4C, D). Similarly, a significant decrease in ubiquitinated AMPK1 was observed in the AMPK1-360aa-overexpressing PC cells compared to the negative control cells (Fig. 4E). The potential ubiquitinated ligases of AMPK1 were further predicted with the Ubirowser database (Fig. 4F). IP revealed that both AMPK1-360aa and AMPK1 could interact with NEDD4, whereas NEDD4L could not interact with AMPK1-360aa (Fig. 4G, H). The immunofluorescence results indicated that AMPK1-360aa and NEDD4 were colocalized (Fig. 4I). Ubiquitination experiments revealed that the level of ubiquitinated AMPK1 was significantly decreased after NEDD4 knockdown, whereas the protein stability of AMPK1 was increased (Additional file 4: Fig. S4F-G). Furthermore, the inhibitory effect of AMPK1-360aa on AMPK1 degradation was reversed by NEDD4 (Fig. 4J). Ubiquitination experiments revealed that AMPK1-360aa prevented the ubiquitination of AMPK1, whereas NEDD4 reversed the inhibitory effect of AMPK1-360aa (Fig. 4K). These results indicate that AMPK1-360aa interacts with NEDD4 to inhibit AMPK1 ubiquitination and degradation.

### AMPK1-360aa competitively binds to NEDD4 and protects AMPK1 from ubiquitination and degradation

**Fig. 5:**
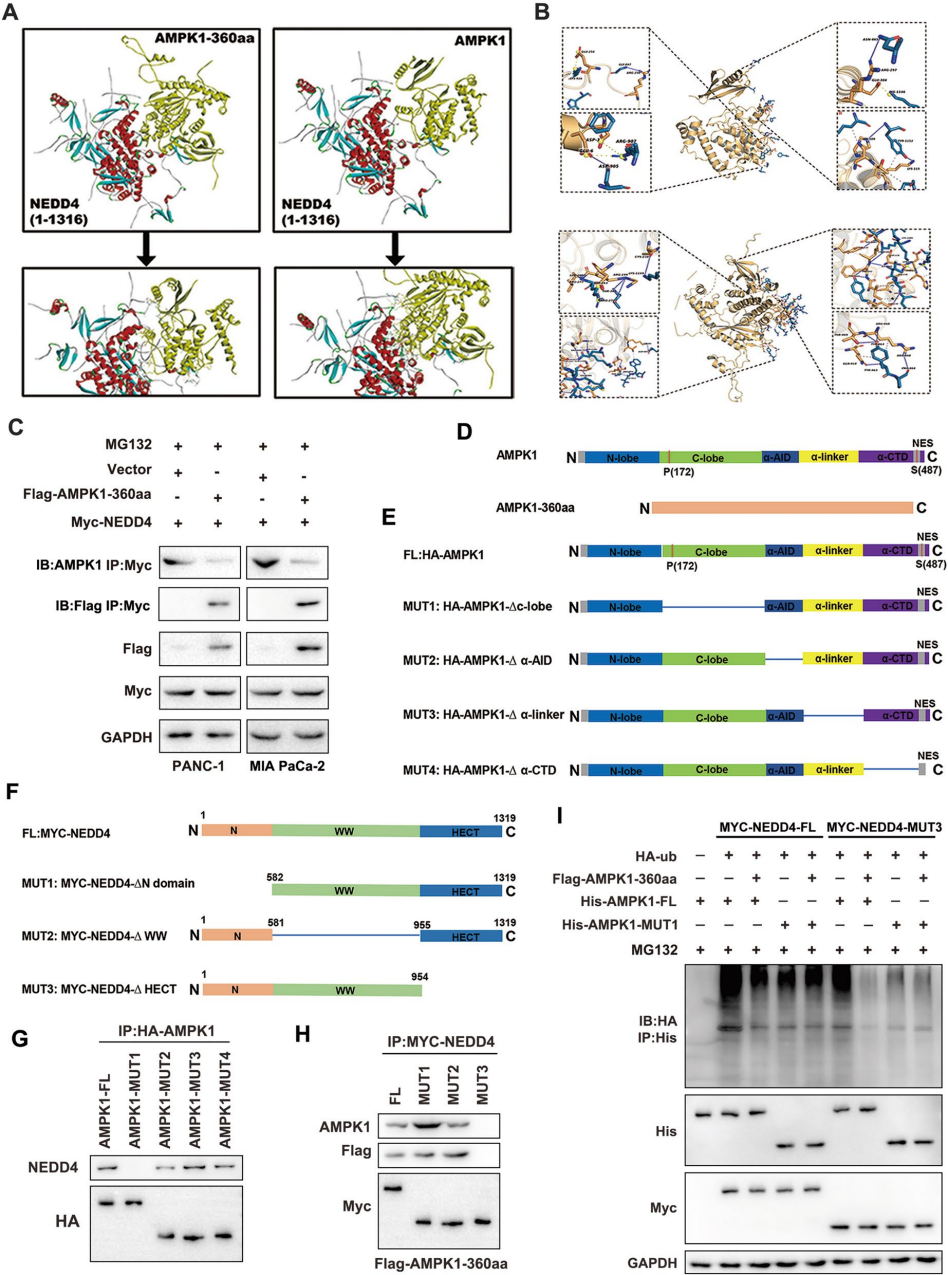
AMPK1-360aa competitively binds NEDD4 and protects AMPK1 from ubiquitination and degradation. **A**, **B** A molecular docking technique was used to demonstrate that AMPK1-360aa and AMPK1 have binding sites for NEDD4. **C** IP was performed to determine whether NEDD4 could bind to AMPK1-360aa. **D** Based on the protein structure of AMPK1, each functional structural domain of the protein was mapped, and the common sequences of AMPK1 and AMPK-360aa were identified. **E** Plasmids were constructed for full-length AMPK1 (FL:HA-AMPK1) and mutants deficient in the C-lobe, α-AID, α-linker and α-CTD. **F** Plasmids were constructed for full-length AMPK1 (FL: MYC-NEDD4) and mutants deficient in the C-lobe, α-AID, α-linker and α-CTD. **G** IP analysis of the interactions of AMPK1 truncation mutants (AMPK1-FL, AMPK1-MUT1, AMPK1-MUT2, AMPK1-MUT3, and AMPK1-MUT4 and NEDD4). **H** IP analysis of the interactions between NEDD4 truncation mutants (MYC-NEDD4-FL, MYC NEDD4-MUT1, MYC-NEDD4-MUT2, and MYC-NEDD4-MUT3) and AMPK1. **I** IP analysis of the effect of NEDD4 and AMPK1-360aa interactions on AMPK1 ubiquitination

The structure of AMPK1-360aa was predicted using I-TASSER software. Docking experiments were then performed with the AMPK1, AMPK1-360aa, and NEDD4 proteins using ZDOCK software, and the most likely docking modes and the major interfacial residues for protein‒protein interactions were determined (Fig. 5A, B). The IP results showed that AMPK1-360aa binds to NEDD4, which is consistent with the results of protein docking (Fig. 5C). The mapping of each functional structural domain of the AMPK1 protein was compared with that of AMPK-360aa, and it was found that there were four main common sequences between the two elements: the C-lobe, α-AID, α-linker and α-CTD (Fig. 5D). To analyze the interaction of the new protein AMPK1-360aa with NEDD4 via shared AMPK1 sequences, we constructed full-length constructs and partial deletion mutants of AMPK1 and NEDD4 (Fig. 5E, F). After transfection of full-length AMPK1 and each mutant plasmid, IP detection of NEDD4 revealed that MUT1 could not bind to NEDD4 (Fig. 5G). In addition, after transfection of full-length NEDD4 and each mutant plasmid, IP detection of NEDD4 revealed that MUT3 could not bind to AMPK1 (Fig. 5H). Therefore, we concluded that the mutated sequence in MUT1 of AMPK1 and the mutated sequence in MUT3 of NEDD4 were critical for binding. In subsequent experiments, 293T cells transfected with the MYC-NEDD4-FL, MYC-NEDD4-MUT3, Flag-AMPK1-360aa, His-AMPK1-FL, and His-AMPK1-MUT1 plasmids were subjected to IP using an anti-ubiquitin antibody. The results showed that the AMPK1 C-lobe structural domain is the binding region of AMPK1-360aa that inhibits AMPK1 ubiquitination and that the HECT domain of NEDD4 is the binding region that promotes AMPK1 ubiquitination (Fig. 5I). These results demonstrate that AMPK1-360aa competitively binds NEDD4 and protects AMPK1 from ubiquitination and degradation.

### AMPK1-360aa/NEDD4/AMPK1 activation mediates proliferation and invasive metastasis in PC cells

**Fig. 6:**
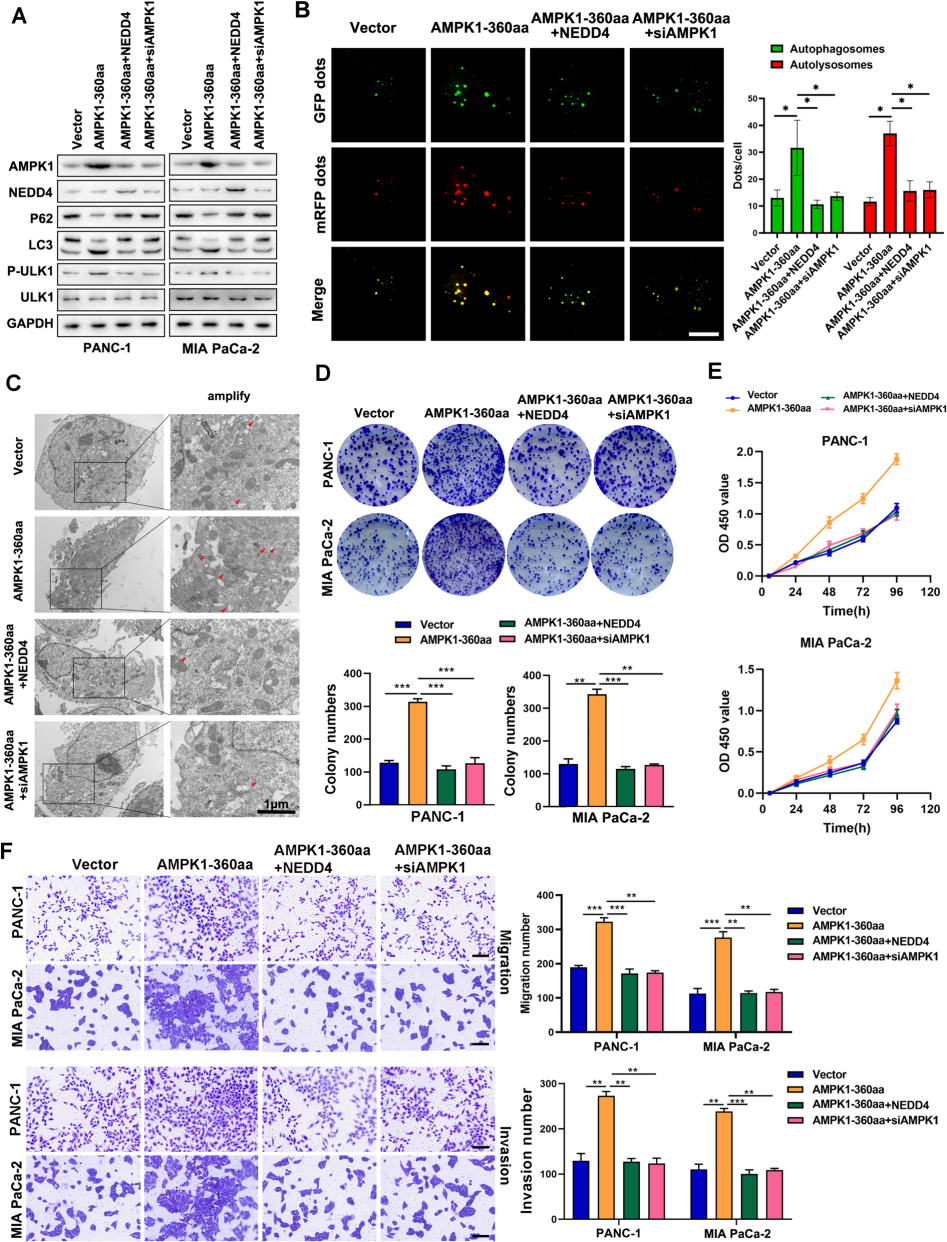
AMPK1-360aa/NEDD4/AMPK1 activation mediates proliferation and invasive metastasis in PC cells. **A** The expression levels of autophagy-related proteins in PC cells in the AMPK1-360aa + siAMPK1, AMPK1-360aa + NEDD4, and empty vector groups were measured using Western blotting. **B** Dual-fluorescence autophagy lentivirus was used to measure autophagy levels in PC cells in the empty vector, AMPK1-360aa + NEDD4, and AMPK1-360aa + siAMPK1 groups. **C** Transmission electron microscopy was used to assess the number of autophagic vesicles in PC cells in the empty vector, AMPK1-360aa + NEDD4, and AMPK1-360aa + siAMPK1 groups. **D**, **E** Plate cloning and the CCK-8 test were used to determine the capacity of PC cells in the AMPK1-360aa + NEDD4, AMPK1-360aa + siAMPK1 and empty vector groups to form colonies and proliferate. **F** Transwell migration and invasion assays were utilized to evaluate the potential of PC cells in the empty vector, AMPK1-360aa + NEDD4, and AMPK1-360aa + siAMPK1 groups to invade and metastasize

To determine the levels of autophagy-related proteins in PC cells, immunoblotting was performed. We discovered that AMPK1-360aa increased autophagy through AMPK1 and that this effect could be reversed by NEDD4 (Fig. 6A). The results of dual-fluorescence autophagy experiments with lentiviruses and transmission electron microscopy observations were consistent with those of the Western blotting experiments (Fig. 6B, C). The results of plate cloning experiments, CCK-8 assays, and Transwell transfer and invasion experiments showed that AMPK1-360aa promoted cell proliferation and metastasis via AMPK1 and that this effect was reversed by NEDD4 (Fig. 6D–F). These results indicate that AMPK1-360aa/NEDD4/AMPK1 activation mediates proliferation and invasive metastasis in PC cells.

### ***CircAMPK1 in CD105***^+^***CAF-derived exosomes promotes PC proliferation and invasive metastasis ***in vivo

**Fig. 7:**
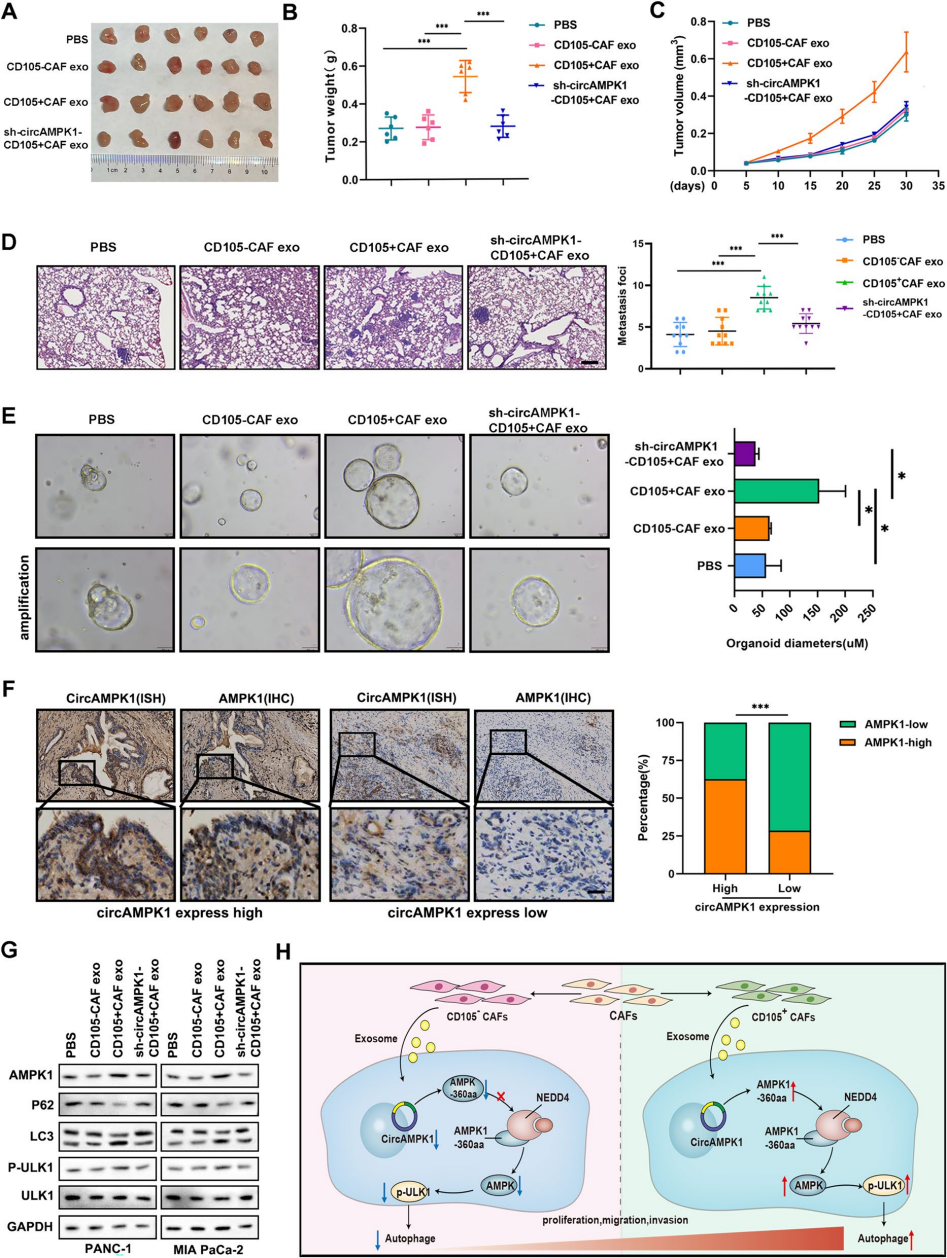
CircAMPK1 in CD105^+^ CAF-derived exosomes promote PC proliferation and invasive metastasis in vivo. **A**–**C** BALB/c nude mice with xenograft tumors show changes in weight and volume following treatment with PBS, CD105^−^ CAF-Exos, CD105^+^ CAF-Exos, or CD105^+^ exosomes from CAFs transfected with sh-circAMPK1. **D** H&E staining analysis of liver metastases from mice treated with PBS, CD105- CAF-Exos, CD105 + CAF-Exos, or CD105^+^ exosomes derived from CAFs transfected with sh-circAMPK1. **E** Statistical graphs showing the proliferation levels in PC organoid models treated with PBS, CD105- CAF-Exos, CD105^+^ CAF-Exos, or CD105^+^ exosomes derived from CAFs transfected with sh-circAMPK1. **F** Statistical analysis of the relationship between the expression of circAMPK1 and the expression of AMPK1 in PC clinical samples. **G** Western blotting analysis of AMPK1-ULK1-autophagy in PC cells following treatment with PBS, CD105^−^ CAF-Exos, CD105^+^ CAF-Exos, or CD105^+^ exosomes from CAFs transfected with sh-circAMPK1. **H** Diagram illustrating the process by which CD105^+^ CAF exosome-derived circAMPK1 modulates PC proliferation and metastasis

A BALB/c nude mouse xenograft tumor nude mouse model and liver metastasis model were constructed, and we found that CD105^+^ CAF-Exos promoted the proliferation and metastasis of tumors and that this procarcinogenic effect was due to circAMPK1 (Fig. 7A–D). To further demonstrate the role of circAMPK1 in CD105^+^ CAF-Exos, we constructed human-derived PC organoids, and the results were consistent with those of the animal experiments (Fig. 7E). In situ hybridization (ISH) and IHC analyses of PC clinical tissue samples revealed that circAMPK1 was positively correlated with AMPK1 expression (Fig. 7F). Western blotting analysis indicated that CD105^+^ CAF-Exos promoted PC cell autophagy via circAMPK1 (Fig. 7G). In summary, the results revealed that circ-AMPK1 in CD105^+^ CAF-Exos encodes AMPK1-360aa and that AMPK1-360aa, NEDD4, and AMPK1 interact with each other to inhibit the ubiquitination of AMPK1 and p-ULK1, thereby promoting autophagy, proliferation, and metastasis in PC cells (Fig. 7H).

## Discussion

CAFs are mesenchymal cells that are abundant in many solid tumors, including pancreatic ductal adenocarcinomas (PDAs)[35]. While altering several aspects of tumor growth, CAFs significantly alter the TME, where their interaction with immune cells is important [36–38]. Previous studies have suggested that the biological functions of CAFs in PC are not fully defined, and they have both pro- and antitumorigenic properties; this lack of clarity is attributed mainly to the high degree of heterogeneity of CAFs in tumors [14]. In addition, the modes of interaction between CAFs and tumor cells are somewhat specific, which explains the uncertainty regarding the biological function of CAFs in tumors [39]. Therefore, clarifying the heterogeneity of CAFs and elucidating their interactions is important for targeting CAFs to effectively fight cancer.

Previous studies have suggested that CD105^+^ CAFs are a key cell subpopulation that promotes the progression of PC; however, the biological functions and regulatory mechanisms of these cells in PC are still unclear. Our group previously reported that exosomes from the CD105^+^ CAF subpopulation in PC tissues significantly increase PC cell proliferation and invasive metastatic ability. Further differential expression analysis of exosomal circRNAs in CD105-/CD105^+^ CAFs revealed that hsa_circ_0003548 (circAMPK1, source gene: AMPK1) was the most significantly upregulated circRNA in CD105^+^ CAFs. CircAMPK1 expression was found to be significantly upregulated in PC tissues, and its expression was strongly associated with poor prognosis. These findings suggest that circAMPK1 may be a key molecule in CD105^+^ CAF-mediated chemotherapy resistance and malignant progression in PC. In recent years, some circRNAs have been found to encode peptides or proteins, and these encoded products often have important biological functions [40]. To further analyze whether circAMPK1 has coding ability, bioinformatics analysis (with circRNADB) was performed. The results revealed that circAMPK1 has an IRES and an ORF and may encode a novel protein with a length of 360 aa. Further studies fully confirmed that circAMPK1 encodes a new protein (AMPK1-360aa) through its ORF; however, the function and structure of this protein are not clear. Related studies have shown that the functions of new proteins encoded by circRNAs may be related to their parental genes; for example, circSMO encodes SMO193aa, which interacts with the full-length parental protein SMO to mediate its cholesterol modification and release from transmembrane receptors to activate the Hedgehog signaling pathway [41]. CircASK1 competitively binds to the upstream kinase AKT, thus inhibiting the phosphorylation of ASK1 and the phosphorylation of AMPK1 by AMPK1-360 aa. The inhibition of ASK1 phosphorylation contributes to increased gemcitabine sensitivity in lung adenocarcinoma [42]. The production of CircGGNBP2, which encodes GGNBP2-184aa, can be induced by IL-6, a protein that has recently been found to interact with STAT3 and promote STAT3 phosphorylation, thus forming an IL-6-STAT3 positive feedback loop that promotes the malignant progression of intrahepatic cholangiocarcinoma [43]. Based on the abovementioned research, we further analyzed the molecular mechanism by which circAMPK1 promotes the upregulation of AMPK1 expression. Our results revealed that both circAMPK1 and AMPK1 bound to NEDD4, whereas NEDD4 mediated the ubiquitination and degradation of AMPK1. Bioinformatics analysis revealed that AMPK1-360aa shares a common sequence with the structure of AMPK1. These results suggest that AMPK1-360aa may competitively bind NEDD4 through its shared C-lobe structure, thus inhibiting the ubiquitination-mediated degradation of AMPK1 and promoting the expression of AMPK1.

Autophagy enhances the initiation and progression of oncogenesis [44, 45]. AMPK is a critical kinase for cellular energy sensing and cell signaling regulation during autophagy. It promotes autophagy mainly by decreasing mTOR activity or increasing the phosphorylation of the autophagy gene Beclin1 [46]. Studies have shown that metastasis and proliferation in many tumors are mediated by the AMPK-dependent TFEB activation pathway, and the inhibition of AMPK or inactivation of mutant TFEB is predicted to be effective in reversing this effect [47]. Studies on autophagy in PC have shown that gemcitabine-resistant PC cells activate autophagy by inducing the expression of PVT1, which promotes the upregulation of ATG14 expression through miR-619-5p, thus allowing the cells to survive chemotherapy [48]. It has been demonstrated that the high expression of AMPK1 can significantly promote the invasive metastatic ability of PC cells by regulating cellular mutations, and its role is closely related to its phosphorylation [49]. The current study showed that tumor cells induce the autophagy signaling pathway in response to PC proliferation and metastasis and that the level of autophagy increases to protect tumor cells and allow them to proliferate. Conversely, the inhibition of autophagy can increase the susceptibility of tumor cells to chemotherapy. Studies in PC have shown that CAFs promote autophagy in tumor cells, thus leading to alanine secretion, which provides energy to tumor cells in low-glycemia microenvironments and is an important factor in the malignant progression of PC [50]. Our findings verify that circAMPK1 within exosomes derived from CD105^+^ CAFs may contribute to PC development by activating autophagy.

## Conclusion

In summary, we identified circAMPK1 in CD105^+^ CAF-Exos as a key molecule that may promote PC progression, and further analysis revealed that circAMPK1 in CD105^+^ CAF-Exos may mediate PC cell proliferation and invasive metastasis through the activation of autophagy. Moreover, circAMPK1 may competitively bind to ubiquitinating enzymes through the encoded protein AMPK1-360aa, which in turn inhibits the ubiquitination-mediated degradation of AMPK1, thus contributing to the upregulation of AMPK1 expression and subsequent induction of cellular autophagy to mediate the malignant progression of PC.

### Supplementary Information

Additional file 1: Fig. S1. CD105^+^ CAFs promote the proliferation, invasion and migration of PC cells. (A) Schematic diagram of CAFs divided into CD105^+^ CAFs and CD105- CAFs and cocultured with PC cells. (B) Plate cloning assay for assessment of the effects of NFs, CD105- CAFs, and CD105^+^ CAFs on PC cell proliferation and the corresponding statistical analyses. (C) Transwell assay for assessment of the effects of NFs, CD105- CAFs, and CD105^+^ CAFs on PC cell invasion and migration and the corresponding statistical analyses. (D) Using a cell scratch test, the effects of NFs, CD105- CAFs, and CD105^+^ CAFs on the migration of PC cells were identified and statistically assessed, as appropriate.

Additional file 2: Fig. S2. CD105^+^ CAF-derived exosomes promote the proliferation, invasion and migration of PC cells. (A) Using a plate cloning experiment, the effects of exosomes released by NFs, CD105^−^ CAFs, and CD105^+^ CAFs on the proliferation of PC cells were monitored and statistically assessed. (B) Using a cell scratch experiment, the effects of exosomes generated by NFs, CD105^−^ CAFs, and CD105^+^ CAFs on the migration of PC cells were examined and statistically assessed.

Additional file 3: Fig. S3. circAMPK1 in exosomes from CD105^+^ CAFs promotes the proliferation, invasion and migration of PC cells. (A) Using a plate cloning experiment, we determined how PBS, sh-Control-Exos, sh-circAMPK1#1-Exos, and sh-circAMPK1#2-Exos affected the proliferation of PC cells. (B) Using Transwell invasion and migration assays, the effects of PBS, sh-Control-Exos, sh-circAMPK1#1-Exos, and sh-circAMPK1#2-Exos on the proliferation of PC cells were evaluated.

Additional file 4: Fig. S4. The AMPK1-360aa/NEDD4 complex inhibits AMPK1 protein degradation and ubiquitination. (A) The effects of circAMPK1-360aa on the levels of AMPK1 mRNA expression were investigated using qRT–PCR. (B) Western blotting analysis of AMPK1 expression in HPDE and PC cells. (C-E) The effects of AMPK1-360aa on the protein expression of AMPK1 were assessed, and the associated statistical analyses were performed after autophagy was blocked using 3MA, Baf, and CQ. (F) The ubiquitination level of AMPK1 in 293T cells transfected with or without siRNA-NEDD4 was analyzed by IP followed by immunoblotting with an anti-ubiquitin antibody. (G) Western blotting analysis of AMPK1 protein stability in PC cells transfected with siRNA-Control or siRNA-NEDD4.

Additional file 5: Table S1. The circRNA sequencing results for CAFs.

Additional file 6: Table S2. Differentially expressed circRNAs in CD105^+^ and CD105^−^ CAFs.

## Data Availability

Not applicable.

## Abbreviations

PC: Pancreatic cancer
CAFs: Tumor-associated fibroblasts
TME: Tumor microenvironment
OS: Overall survival
DFS: Disease-free survival
CircRNA: Circular RNA
H&E: Hematoxylin and eosin
IRES: Internal ribosome entry site
