# Supplementary figures and images for: CD105+CAF-derived exosomes CircAMPK1 promotes pancreatic cancer progression by activating autophagy
Source: Exp Hematol Oncol. 2024 Aug 5;13:79. doi: 10.1186/s40164-024-00533-3 (PMC11301837; doi:10.1186/s40164-024-00533-3)

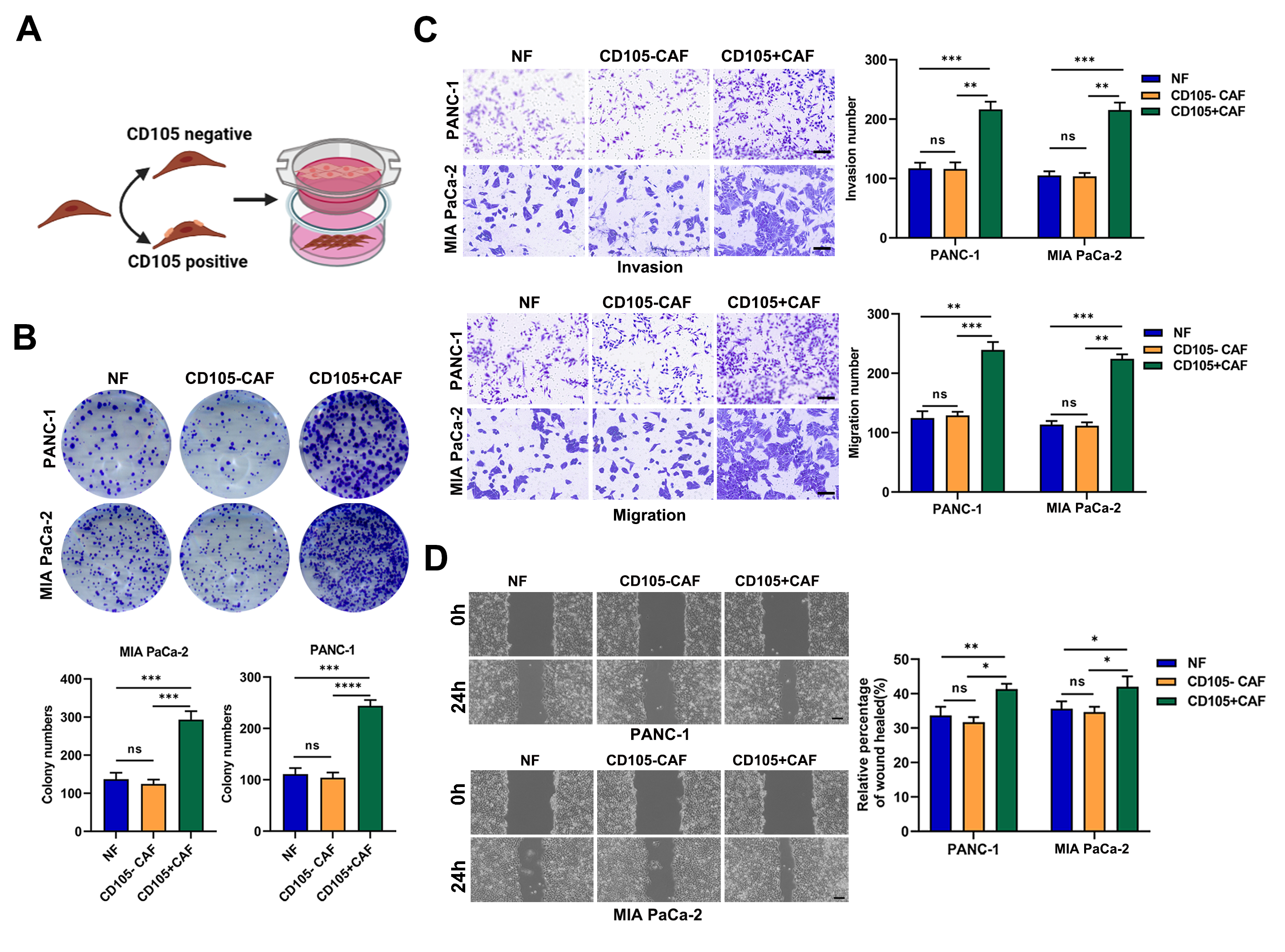

Supplement: Supplementary file 1 — Additional file 1: Fig. S1. CD105+ CAFs promote the proliferation, invasion and migration of PC cells. (A) Schematic diagram of CAFs divided into CD105+ CAFs and CD105- CAFs and cocultured with PC cells. (B) Plate cloning assay for assessment of the effects of NFs, CD105- CAFs, and CD105+ CAFs on PC cell proliferation and the corresponding statistical analyses. (C) Transwell assay for assessment of the effects of NFs, CD105- CAFs, and CD105+ CAFs on PC cell invasion and migration and the corresponding statistical analyses. (D) Using a cell scratch test, the effects of NFs, CD105- CAFs, and CD105+ CAFs on the migration of PC cells were identified and statistically assessed, as appropriate. [file 40164_2024_533_MOESM1_ESM.tif]

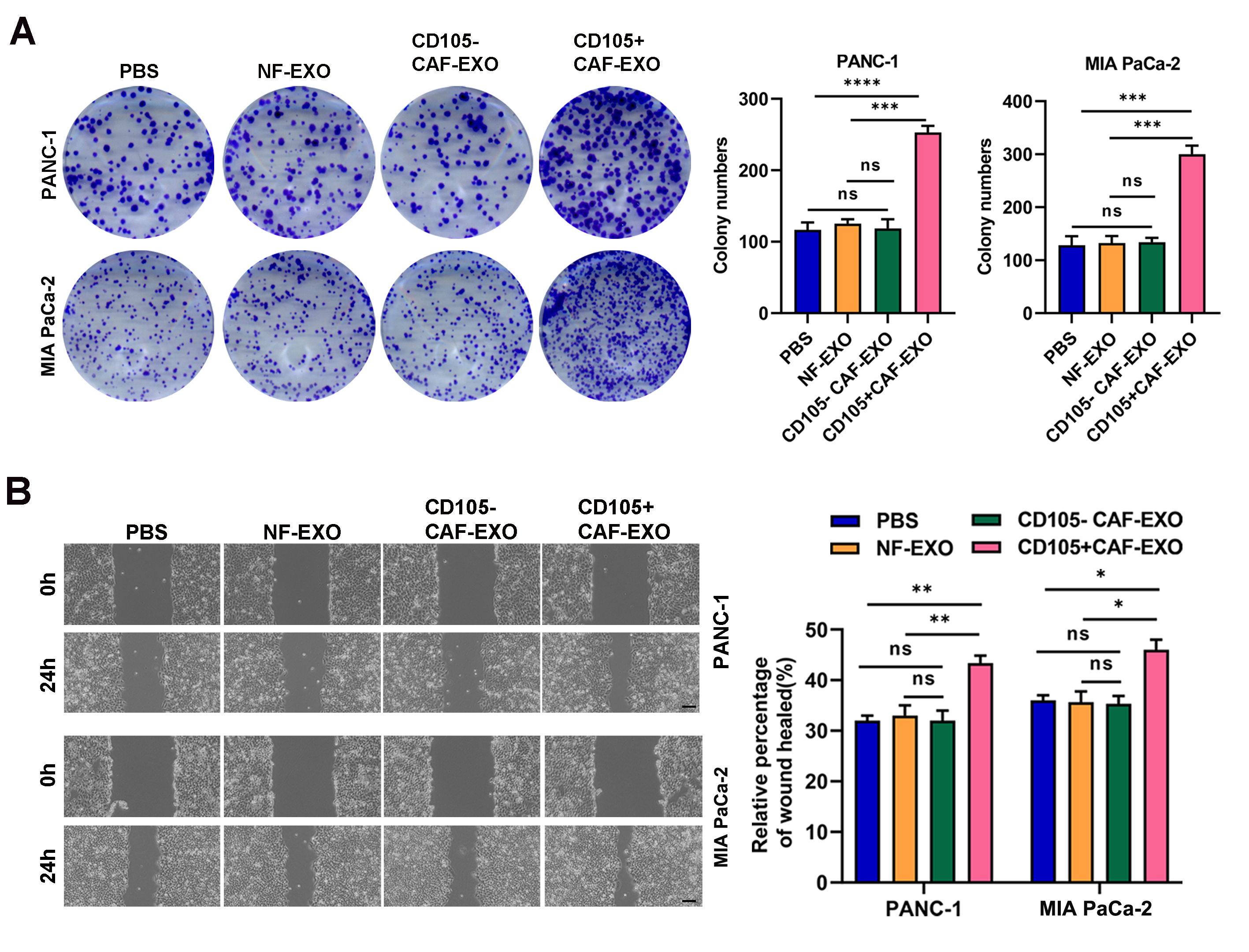

Supplement: Supplementary file 2 — Additional file 2: Fig. S2. CD105+ CAF-derived exosomes promote the proliferation, invasion and migration of PC cells. (A) Using a plate cloning experiment, the effects of exosomes released by NFs, CD105− CAFs, and CD105+ CAFs on the proliferation of PC cells were monitored and statistically assessed. (B) Using a cell scratch experiment, the effects of exosomes generated by NFs, CD105− CAFs, and CD105+ CAFs on the migration of PC cells were examined and statistically assessed. [file 40164_2024_533_MOESM2_ESM.tif]

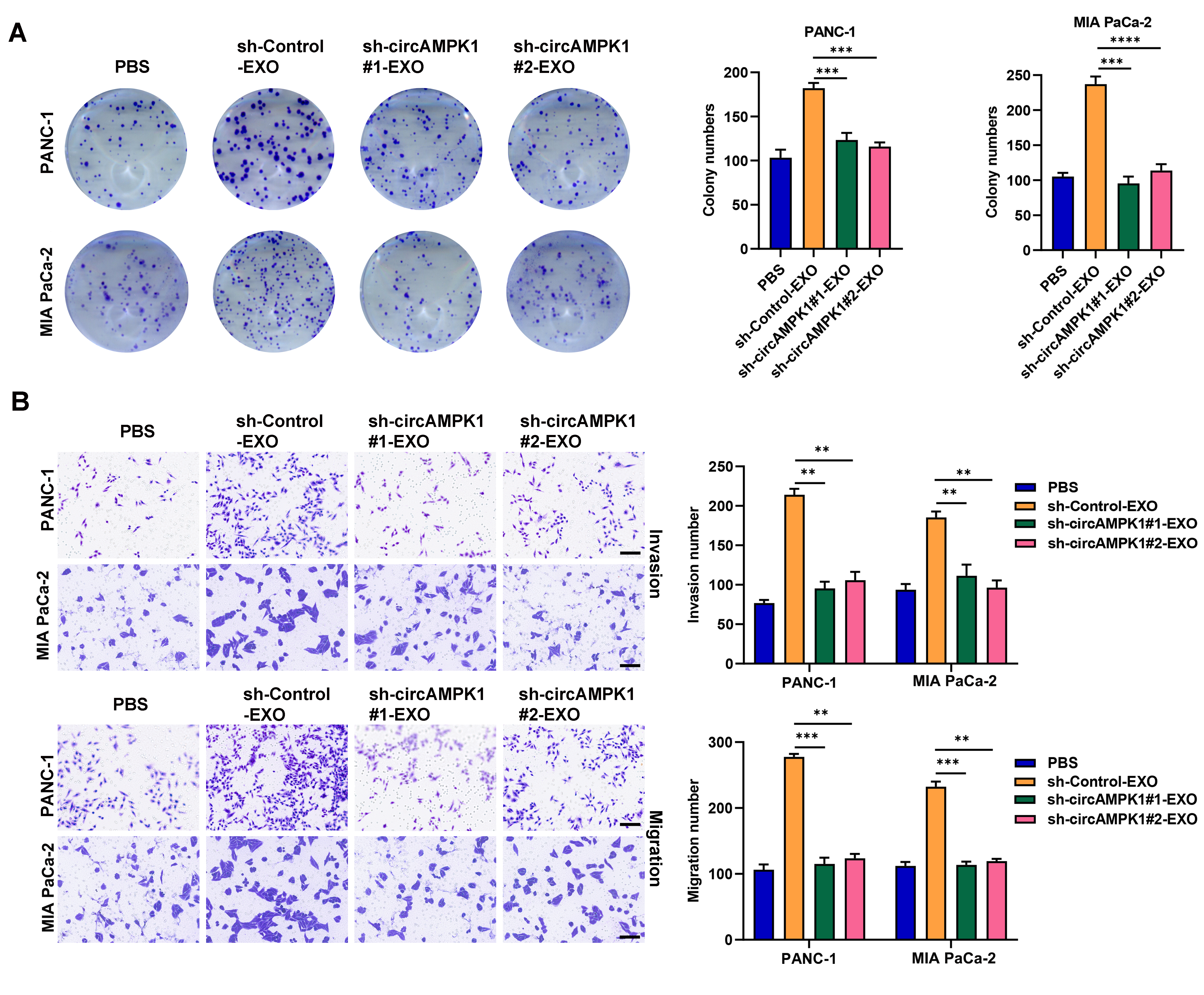

Supplement: Supplementary file 3 — Additional file 3: Fig. S3. circAMPK1 in exosomes from CD105+ CAFs promotes the proliferation, invasion and migration of PC cells. (A) Using a plate cloning experiment, we determined how PBS, sh-Control-Exos, sh-circAMPK1#1-Exos, and sh-circAMPK1#2-Exos affected the proliferation of PC cells. (B) Using Transwell invasion and migration assays, the effects of PBS, sh-Control-Exos, sh-circAMPK1#1-Exos, and sh-circAMPK1#2-Exos on the proliferation of PC cells were evaluated. [file 40164_2024_533_MOESM3_ESM.tif]

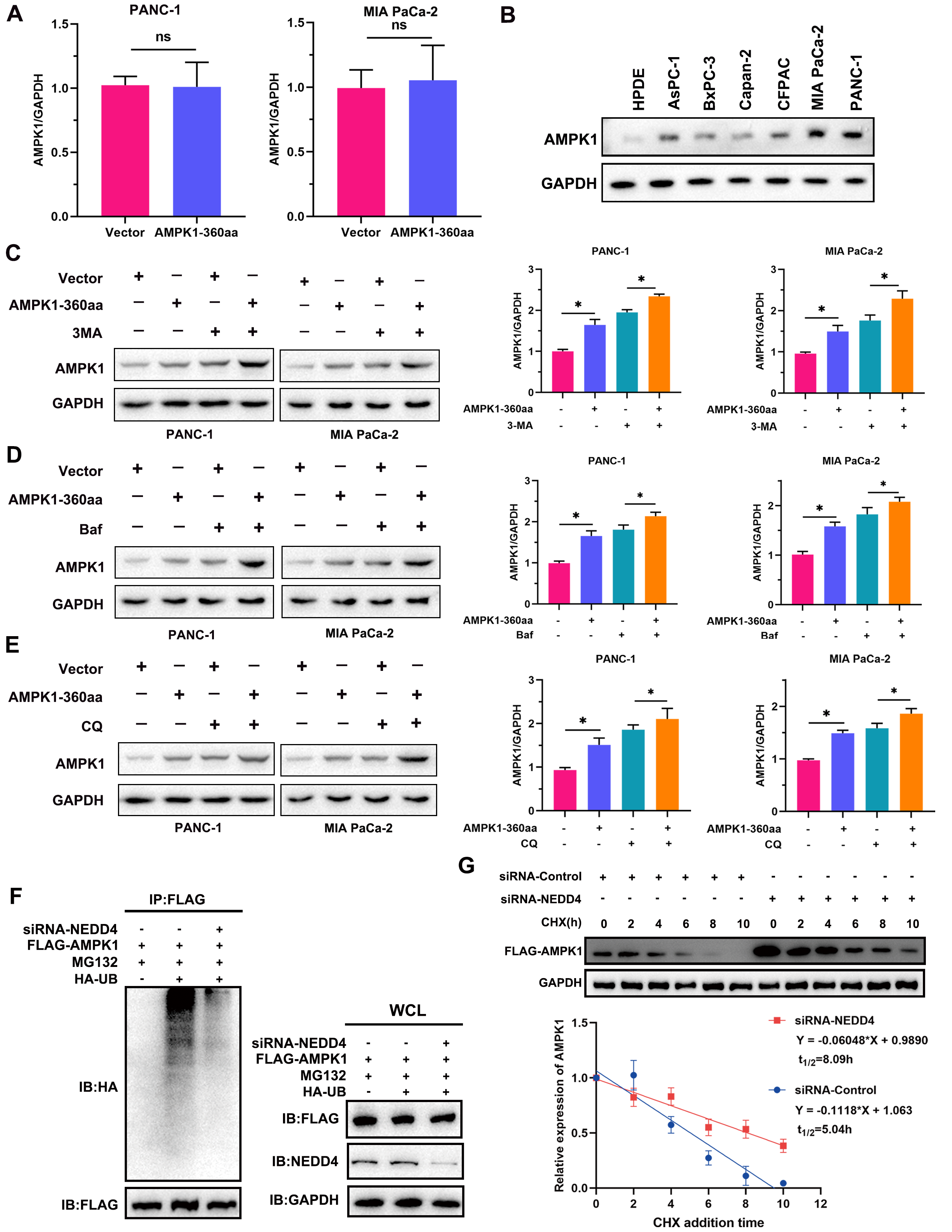

Supplement: Supplementary file 4 — Additional file 4: Fig. S4. The AMPK1-360aa/NEDD4 complex inhibits AMPK1 protein degradation and ubiquitination. (A) The effects of circAMPK1-360aa on the levels of AMPK1 mRNA expression were investigated using qRT–PCR. (B) Western blotting analysis of AMPK1 expression in HPDE and PC cells. (C-E) The effects of AMPK1-360aa on the protein expression of AMPK1 were assessed, and the associated statistical analyses were performed after autophagy was blocked using 3MA, Baf, and CQ. (F) The ubiquitination level of AMPK1 in 293T cells transfected with or without siRNA-NEDD4 was analyzed by IP followed by immunoblotting with an anti-ubiquitin antibody. (G) Western blotting analysis of AMPK1 protein stability in PC cells transfected with siRNA-Control or siRNA-NEDD4. [file 40164_2024_533_MOESM4_ESM.tif]
